# 220. Safety and Immunogenicity of mRNA-1018, a candidate vaccine for the prevention of H5N1 pandemic influenza, in healthy adults ≥18 years of age in a dose-ranging Phase 1/2 clinical study

**DOI:** 10.1093/ofid/ofaf695.078

**Published:** 2026-01-11

**Authors:** Natalia V Voge, Catherine A Cosgrove, Andrei Avanesov, Kingston Kang, Angela Choi, Dondi Rust, Kyle Kellinghaus, Yoonyoung Park, Riya Joshi, Agi Buchanan, Jean Hu-Primmer, Alan Embry, Raffael Nachbagauer, Brett Leav

**Affiliations:** Moderna, Cambridge, Massachusetts; St Georges University Hospitals, London, England, United Kingdom; Moderna, Inc, Cambridge, Massachusetts; Moderna, Cambridge, Massachusetts; Moderna, Cambridge, Massachusetts; Moderna, Cambridge, Massachusetts; Moderna, Cambridge, Massachusetts; Moderna, Cambridge, Massachusetts; Moderna, Cambridge, Massachusetts; Moderna, Inc., Cambridge, Massachusetts; Moderna, Cambridge, Massachusetts; Moderna Therapeutics, Cambridge, Massachusetts; Moderna, Inc, Cambridge, Massachusetts; Moderna, Cambridge, Massachusetts

## Abstract

**Background:**

Influenza A viruses pose a persistent pandemic threat due to their zoonotic potential and capability of antigenic shift, enabling the emergence of novel strains with pandemic potential.

**Methods:**

This parallel design, dose-ranging, blinded study, assessed the safety and immunogenicity of three dose-levels (12.5, 25 and 50 µg) of mRNA-1018. This pre-pandemic vaccine, which encodes for the hemagglutinin (HA) of H5-A/chicken/Ghana/2021, was given as two doses 21 days apart to 304 healthy adults (> 18 to < 65 and > 65 years of age). Immune responses were assessed by hemagglutination inhibition (HAI) and microneutralization (MN) at multiple time points during the 6-month study.

**Results:**

Most local and systemic solicited adverse reactions (AR) were Grade 1–2, and the most common AR were injection site pain, fatigue, and headache. Grade 3 AR were infrequent (< 5%), and no Grade 4 events occurred. The rates of solicited AR following the second dose were comparable to the first, with no notable increase in severity. One related SAE (syncope) was reported.

Three weeks after the second dose, the percentage of participants in all dose groups with HAI titers ≥ 1:40 (defined as seroprotection or SP) were 97.8% (95% confidence interval [95%CI]: 95.4, 99.2). The percentage with seroconversion (SC) by HAI (defined as post-vaccination titer ≥ 1:40 if baseline is < 1:10 or a 4-fold or greater rise if baseline is ≥ 1:10) was 97.1% (95%CI: 94.4–98.7). Immune responses were detected early across all dose groups and remained detectable at study end. Three weeks after the first dose, 79.5% (95%CI: 74.3, 84.1) of participants had achieved SP and 78.1% (95%CI: 72.8, 82.8) met criteria for SC. Six months after the second dose, 70.6 % (95%CI: 64.8, 76.0) maintained SP levels. Titers measured by both HAI and microneutralization (MN) assays increased with increasing dose of mRNA-1018.

**Conclusion:**

Across all dose levels, mRNA-1018 was safe, well-tolerated, and demonstrated rapid and persistent immune response which are considered key attributes of a pandemic vaccine.

**Disclosures:**

All Authors: No reported disclosures

